# Perioperatives anästhesiologisches Management der postmortalen Organspende in Deutschland – Eine prospektive Querschnittstudie mittels Online-Fragebogen

**DOI:** 10.1007/s00101-026-01647-5

**Published:** 2026-01-28

**Authors:** Tobias Piegeler, Jan S. Englbrecht, Martin Söhle, Madeleine Ordnung, Klaus Hahnenkamp, Svitlana Ziganshyna

**Affiliations:** 1 Klinik und Poliklinik für Anästhesiologie und Intensivtherapie, Universitätsklinikum Leipzig, Liebigstraße 20, 04103 Leipzig, Deutschland; 2 Klinik für Anästhesiologie, operative Intensivmedizin und Schmerztherapie, Universitätsklinikum Münster, Münster, Deutschland; 3 Klinik für Anästhesiologie und Operative Intensivmedizin, Universitätsklinikum Bonn, Bonn, Deutschland; 4 Pädiatrische Epidemiologie, Klinik und Poliklinik für Kinder- und Jugendmedizin, Universitätsklinikum Leipzig, Leipzig, Deutschland; 5 Klinik für Anästhesie, Intensiv‑, Notfall-, und Schmerzmedizin, Universitätsmedizin Greifswald, Greifswald, Deutschland; 6 Stabsstelle Transplantationsbeauftragte, Universitätsklinikum Leipzig, Leipzig, Deutschland; 7 Kommission Organspende und Organtransplantation der Deutschen Gesellschaft für Anästhesiologie und Intensivmedizin, Nürnberg, Deutschland

**Keywords:** Organspende, Anästhesiologie, Organprotektion, Transplantation, Behandlungsergebnis, Organ donation, Anesthesiology, Organ protection, Transplantation, Outcome

## Abstract

**Hintergrund:**

Im Gegensatz zur intensivmedizinischen Therapie existieren bisher keine Handlungsempfehlungen zum anästhesiologischen Management postmortaler Organspenderinnen und Organspender in Deutschland. Zudem ist unbekannt, wie die anästhesiologische Versorgung in den Entnahme-Krankenhäusern durchgeführt wird.

**Ziel der Arbeit:**

Erfassung der Praxis des anästhesiologischen Managements der postmortalen Organspende.

**Methoden:**

Prospektive Querschnittstudie (DRKS00033627) mittels anonymisierter Online-Umfrage unter den Mitgliedern der Deutschen Gesellschaft für Anästhesiologie und Intensivmedizin sowie den bei der Deutschen Stiftung für Organtransplantation registrierten Transplantationsbeauftragten. Neben demografischen Angaben und der eigenen Erfahrung in der Betreuung von Organspenderinnen und Organspendern wurde das konkrete anästhesiologische Vorgehen während eines solchen Eingriffs erfragt.

**Ergebnisse:**

Es wurden insgesamt 951 Fragebogen aus allen stationären Versorgungsstufen ausgewertet (28 % Grund‑/Regelversorger, 29 % Schwerpunktversorger, 17 % Maximalversorger, 24 % Universitätskliniken, 1 % Sonstige). Die Antworten zum konkreten Vorgehen, insbesondere bezüglich der eingesetzten Medikamente und der Indikation zur Transfusion von Erythrozytenkonzentraten, waren insgesamt heterogen. In einer multivariaten logistische Regressionsanalyse zeigte sich, dass neben der Versorgungsstufe der Kliniken v. a. die Absolvierung des Curriculums „Transplantationsbeauftragter Arzt“ einen Einfluss auf die Verabreichung spezifischer (potenziell organprotektiver) Medikamente haben könnte.

**Diskussion:**

Die anästhesiologische Betreuung zur postmortalen Organspende wird in Deutschland aktuell sehr unterschiedlich gehandhabt. Die Erstellung von Handlungsempfehlungen erscheint daher angezeigt.

**Zusatzmaterial online:**

Die Online-Version dieses Beitrags (10.1007/s00101-026-01647-5) enthält das im Text erwähnte Zusatzmaterial. Bitte scannen Sie den QR-Code.

## Abstract (en)

**Background:**

In contrast to intensive care, no specific recommendations regarding the anesthesiological management during organ retrieval exist in Germany. Additionally, it remains unknown how anesthesiological care is performed in the respective hospitals hosting the donor. To address this knowledge gap, this study aimed to assess current anesthesiological practice in this respect.

**Aim:**

To document the practice of anesthesiological management in postmortem organ donation.

**Methods:**

A prospective cross-sectional study was conducted among the members of the German Society for Anesthesiology and Intensive Care Medicine and the donation coordinators registered with the German Organ Procurement Organization using an anonymized online questionnaire. Besides demographic data and individual experience in managing organ donors, the actual anesthesiological approach during organ procurement was surveyed. Prior to initiation the study was registered with the German Clinical Trials Register (DRKS00033627). Statistical analysis was conducted using descriptive methods as well as multivariate logistic regression.

**Results:**

A total of 951 questionnaires from respondents at hospitals with different levels of care could be analyzed (28% primary, 29% secondary, 17% tertiary, 24% university hospitals, 1% others). Responses regarding the actual approach, especially the use of different medications were heterogeneous. A multivariate logistic regression revealed that the hospital’s level of care and the successful completion of the organ donation coordinator curriculum might affect the administration of specific (potentially organ protective) medications, such as dopamine or glucocorticoids. Answers regarding the hemodynamic management and indications for the transfusion of blood components in organ donors were also diverse: 51% specifically indicated to aim for a mean arterial pressure of at least 65–69 mm Hg during the procedure, 16% stated the threshold to be below these values and another 28% aimed for higher values. In cases of hemodynamic stability 47% of the respondents would initiate the transfusion of erythrocyte concentrates at hemoglobin levels below 4.3 mmol/l (7.0 g/dl), while 50% would already administer them below 5.0 mmol/l (8.0 g/dl), when there is concomitant hemodynamic instability. Another 11% indicated that in their opinion a transfusion would not be indicated at all during organ procurement.

**Conclusion:**

The anesthesiological management of postmortem organ donation in Germany currently varies significantly, which might at least in part be due to the lack of specific guidelines or recommendations. The results of the current study represent the first scientific approach regarding this issue in Germany and they underline the importance of education in this respect, especially in hospitals with lower levels of care and less frequent exposure to organ donations. The development of practice recommendations appears warranted in order to standardize the anesthesiological approach regarding organ donation, thus extending and optimizing organ-protective measures and improving outcome after solid organ transplantation.

## Einleitung

Die postmortale Organspende ist essenzieller Bestandteil der modernen Transplantationsmedizin und bietet betroffenen Personen mit terminalem Organversagen eine lebensrettende Therapieoption. Der Erfolg von Organtransplantationen hängt maßgeblich ab von der Qualität der gespendeten Organe, die wiederum durch das organprotektive Management des Organspenders beeinflusst wird. Dieses Management ist ein kontinuierlicher Prozess, der auf der Intensivstation beginnt und im OP fortgesetzt wird. Während für die intensivmedizinische Versorgung des Organspenders internationale Empfehlungen und Leitlinien existieren, fehlt es an einem Konsens für das anästhesiologische Management während der Organentnahme [15]. Zudem basieren die intensivmedizinischen Empfehlungen auf unzureichender Evidenz und stützen sich überwiegend auf Expertenmeinungen, Registerstudien und retrospektive Analysen [14, 16].

Das anästhesiologische Management birgt dabei besondere Herausforderungen, die sich deutlich von der Versorgung lebender Patienten unterscheiden [32]. Die Komplexität ergibt sich u. a. aus den pathophysiologischen Veränderungen im Rahmen des irreversiblen Hirnfunktionsausfalls und der Notwendigkeit, die – intensivmedizinisch begonnenen – organprotektiven Maßnahmen während der Entnahme adäquat fortzuführen. Trotz der zentralen Rolle der intraoperativen Betreuung der Organspendenden für die Qualität der zu entnehmenden Organe führt die aktuelle Situation, ohne einheitliche nationale Standards [15], zu einer erheblichen Variabilität in der klinischen Praxis und verdeutlicht die Notwendigkeit eines strukturierten Ansatzes zur Harmonisierung der Versorgung [8].

Ziel der vorliegenden Studie war es daher, bestehende Variationen und Optimierungsmöglichkeiten des anästhesiologischen Managements der postmortalen Organspende zu identifizieren, um eine Grundlage für die Entwicklung nationaler Handlungsempfehlungen zu schaffen. Um die aktuelle Praxis dieses Bereichs des ärztlichen Handelns in Deutschland zu erfassen, wurde eine anonymisierte Online-Umfrage unter den Mitgliedern der Deutschen Gesellschaft für Anästhesiologie und Intensivmedizin (DGAI) sowie den bei der Deutschen Stiftung für Organtransplantation (DSO) registrierten Transplantationsbeauftragten (TxB) durchgeführt. Die Ergebnisse der vorliegenden Studie stellen somit den ersten Schritt in Richtung der Entwicklung nationaler, standardisierter Handlungsempfehlungen dar, was einen bedeutenden Beitrag zur Verbesserung der Versorgungsqualität sowie zur Optimierung der Ergebnisse von Organtransplantationen leisten könnte.

## Methoden

### Fragebogen

Die Umfrage zur aktuellen Praxis des anästhesiologischen Managements der postmortalen Organspende wurde im Rahmen einer prospektiven Querschnittstudie mittels eines Online-Fragebogens durchgeführt. Dieser erfasste demografische Charakteristika der Befragten, organisatorische Aspekte der Organspende in den jeweiligen Einrichtungen sowie medizinische Aspekte, darunter anästhesiologisch relevante Medikamentengaben sowie das hämodynamische Management während der postmortalen Organspende. Der Fragebogen wurde über den E‑Mail-Verteiler an die Mitglieder der Deutschen Gesellschaft für Anästhesiologie und Intensivmedizin (DGAI) sowie an die bei der Deutschen Stiftung für Organtransplantation (DSO) registrierten TxB verteilt (insgesamt 11.356 E‑Mail-Adressen, 10.009 Mitglieder der DGAI, 1347 DSO-TxB). Die Teilnahme war freiwillig und für insgesamt 4 Wochen möglich; eine Erinnerung erfolgte nach 2 Wochen. Alle Daten wurden anonymisiert erhoben. Der Fragebogen wurde mit der evasys Software (evasys V9.1, evasys GmbH, Lüneburg, Deutschland) erstellt.

Die Studie wurde vorab mit dem zuständigen Datenschutzbeauftragten der Medizinischen Fakultät der Universität Leipzig abgestimmt, und die Ethik-Kommission der Medizinischen Fakultät der Universität Leipzig hatte festgestellt, dass keine berufsrechtliche und -ethische Beratungspflicht gemäß § 15 Berufsordnung der Sächsischen Landesärztekammer bestand. Die Studie wurde beim Deutschen Register Klinischer Studien (DRKS) registriert (DRKS00033627).

### Statistische Analyse

**Abb. 1:**
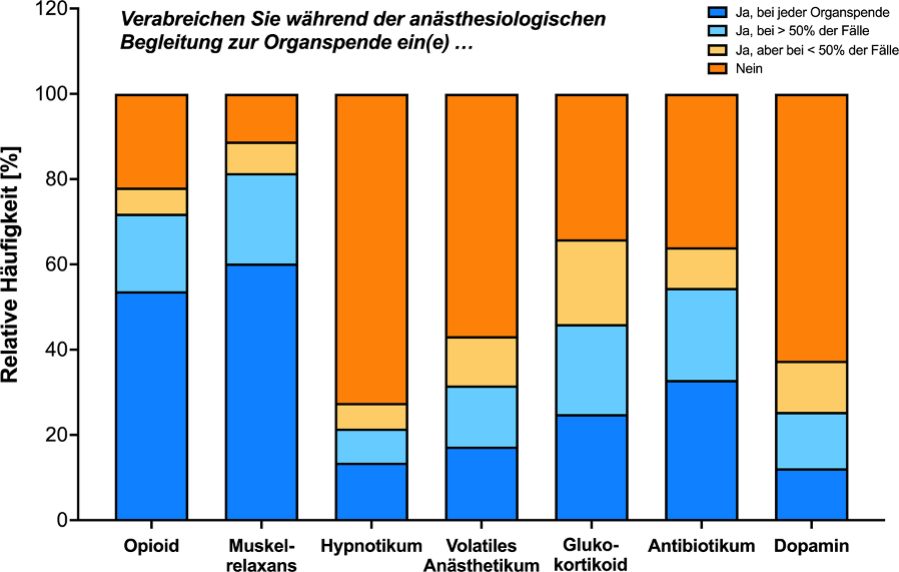
Relative Häufigkeit der Medikamentengabe (in Prozent) während einer postmortalen Organspende, kategorisiert nach den verschiedenen Antwortmöglichkeiten im Rahmen des Online-Fragebogens („Ja, bei jeder Organspende“: *blau*; „Ja, bei > 50 % der Fälle“: *hellblau*; „Ja, aber bei < 50 % der Fälle“: *blass-orange*; „Nein“: *orange*)

**Abb. 2:**
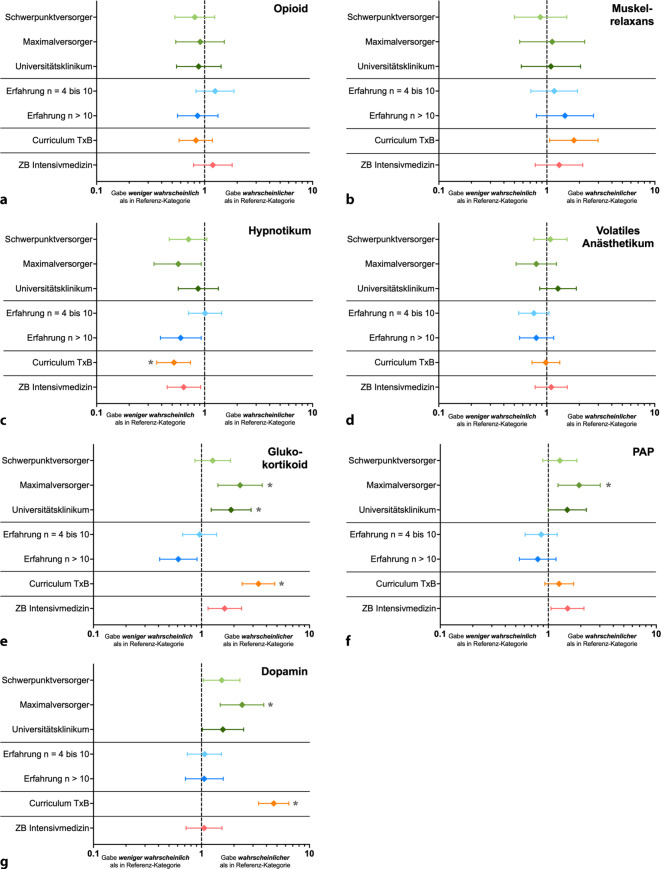
Forest-Plots zur Darstellung der Ergebnisse der logistischen Regressionsanalyse zur Evaluation des Einflusses verschiedener Faktoren (Versorgungsstufe, Erfahrung in der Betreuung von Organspenden, Absolvierung des Curriculums zum Transplantationsbeauftragten (TxB), Vorhandensein der Zusatzbezeichnung (ZB) Intensivmedizin) auf die Wahrscheinlichkeit der Gabe der verschiedenen anästhesiologisch relevanten Medikamente während einer postmortalen Organspende (**a** Opioid, **b** Muskelrelaxans, **c** (i.v.-)Hypnotikum, **d** volatiles Anästhetikum, **e** Glukokortikoid, **f** perioperative Antibiotikaprophylaxe (*PAP*), **g** Dopamin). Dargestellt sind die jeweiligen Wahrscheinlichkeiten der Gabe (Odds ratio, *Raute*) mit dem korrespondierenden 95 %igen Konfidenzintervall. *X‑Achse* logarithmisch. Referenzkategorien: Versorgungsstufe: Grund‑/Regelversorger; Erfahrung: *n* = 0–3; Curriculum: Nicht absolviert; ZB Intensivmedizin: Nicht vorhanden. *p < 0,005

Die Auswertung der Umfrage erfolgte zunächst deskriptiv durch die Ermittlung der relativen Häufigkeiten einzelner Antwortkategorien (Tab. 1 und 2; Abb. 1, Zusatzmaterial online: ESM 1). Anschließend wurde mittels multivariater logistischer Regression (Abb. 2; Tab. 3 und Zusatzmaterial online: ESM 2) exemplarisch untersucht, ob die Gabe von Medikamenten (Opioid, Muskelrelaxans, (i.v.-)Hypnotikum, volatiles Anästhetikum, Glukokortikoid, perioperative Antibiotikaprophylaxe (PAP), Dopamin) oder die Indikationsstellung zur Transfusion während der postmortalen Organspende von demografischen Charakteristika der Befragten (Versorgungsstufe des Entnahmekrankenhauses, Erfahrung in der Durchführung von Organspenden, Absolvieren des Curriculums zum TxB sowie Vorhandensein der Zusatzweiterbildung (ZB) Intensivmedizin) beeinflusst wird. Diese zusätzliche Analyse wurde durchgeführt, da bislang weder auf nationaler noch auf internationaler Ebene Daten zum anästhesiologischen Management während der Organspende vorliegen. Die Ergebnisse könnten Hinweise liefern auf besonders relevante Aspekte, die bei der Entwicklung von Handlungsempfehlungen berücksichtigt werden sollten.

**Tab. 1:** Demografische Daten der Befragten (*N* = 949).

Variable	Anteil (%)
*Geschlecht *(weiblich/männlich/divers/keine Angabe)	32/65/0,3/2,1
*Berufserfahrung *(inkl. Weiterbildung)	
weniger als 5 Jahre	1
5–10 Jahre	10
mehr als 10 Jahre	89
*Versorgungsstufe*	
Grund- und Regelversorger	28
Schwerpunktversorger	29
Maximalversorger	17
Universitätsklinik	24
*Ärztliche Position*	
Chefärztliche, direktorische Leitung	15
Oberärztliche Position	61
Fachärztliche Position	22
In Weiterbildung	2
*Aktive Beteiligung *an postmortalen Organentnahmen	
Keine	5
1–3 Entnahmen	28
4–10 Entnahmen	38
11–20 Entnahmen	13
Mehr als 20 Entnahmen	16
Tätigkeit als *Transplantationsbeauftragter *(ja/nein)	33/67
Absolvieren des *Curriculums „Transplantationsbeauftragter Arzt“* (ja/nein)	35/65
Vorhandensein der *Zusatzbezeichnung Intensivmedizin* (ja/nein)	77/23

**Tab. 2:** Hämodynamisches Management während postmortaler Organspende (*n* = 944).

*Frage*	Anteil (%)
Verwendung eines *erweiterten hämodynamischen Monitorings* (z. B. invasive Blutdruck-Messung, Pulskonturanalyse, Pulmonalarterienkatheter etc.)	
Bei jeder Organspende	59
Bei der überwiegenden Zahl der Spenden	24
Bei weniger als 50 % der Organspenden	5
Keines	12
Angestrebter *mittlerer arterieller Blutdruck* (Minimum/Untergrenze)	
50–59 mm Hg	1
60–64 mm Hg	15
65–69 mm Hg	51
70–79 mm Hg	25
Mehr als 80 mm Hg	3
Keine festgelegte Grenze	5
*Katecholamin* der ersten Wahl zur Erhöhung des *peripheren Gefäßwiderstands*	
Noradrenalin	97
Adrenalin	1
Cafedrin Theodrenalin (Akrinor®)	1
Andere	1
*Katecholamin* der ersten Wahl zur Erhöhung der *Inotropie des Herzens*	
Dobutamin	83
Adrenalin	12
Dopamin	3
Andere	2
Hb-Wert zur *Indikation einer Transfusion* eines Erythrozytenkonzentrats bei einem *kreislaufstabilen* Organspender	
< 3,7 mmol/l (6,0 g/dl)	21
< 4,3 mmol/l (7,0 g/dl)	47
< 5,0 mmol/l (8,0 g/dl)	18
< 5,6 mmol/l (9,0 g/dl)	2
< 6,2 mmol/l (10,0 g/dl)	2
Transfusion nicht indiziert bei Organentnahme	11
Hb-Wert zur *Indikation einer Transfusion* eines Erythrozytenkonzentrates bei einem *kreislaufinstabilen* Organspender	
< 3,7 mmol/l (6,0 g/dl)	5
< 4,3 mmol/l (7,0 g/dl)	18
< 5,0 mmol/l (8,0 g/dl)	50
< 5,6 mmol/l (9,0 g/dl)	16
< 6,2 mmol/l (10,0 g/dl)	8
Transfusion nicht indiziert bei Organentnahme	4

**Tab. 3:** Ergebnisse der multiplen logistischen Regressionsanalyse hinsichtlich der Indikationsstellung zur Transfusion.

Transfusionsindikation/Einflussfaktor	Kreislaufstabil: < 4,3 mmol/l	Kreislaufinstabil: < 5,0 mmol/l
*Versorgungsstufe*		
Schwerpunktversorger (OR (95%-KI))	1,28 (0,84–1,97)	0,87 (0,57–1,32)
Maximalversorger (OR (95%-KI))	1,51 (0,89–2,57)	0,65 (0,38–1,09)
Universitätsklinikum (OR (95%-KI))	1,32 (0,82–2,14)	0,83 (0,52–1,31)
*Erfahrung*		
*n* = 4 bis 10 (OR (95%-KI))	0,91 (0,60–1,39)	1,18 (0,80–1,75)
*n* > 10 (OR (95%-KI))	0,69 (0,44–1,08)	1,17 (0,75–1,83)
*Curriculum TxB* (OR (95%-KI))	0,99 (0,70–1,42)	0,74 (0,51–1,06)
*Zusatzbezeichnung Intensivmedizin *(OR (95%-KI))	0,90 (0,57–1,41)	0,86 (0,58–1,29)

*OR* Odds ratio, *KI* Konfidenzintervall. Verwendete Referenzkategorien: Versorgungsstufe: Grund- und Regelversorger, Erfahrung Organspende: *n* = 0 bis 3, Curriculum: nicht vorhanden/absolviert, Zusatzbezeichnung Intensivmedizin: nicht vorhanden/absolviert.

Für die multivariate logistische Regression (Abb. 2 und Zusatzmaterial online: ESM 2) wurden die Antwortkategorien der Variable Medikamentengabe dichotomisiert („Ja“, „Nein“). Im Fall der Frage nach der Transfusionsindikation erfolgte ebenfalls eine Dichotomisierung: Hier wurde untersucht, inwieweit die Befragten die Indikation zur Transfusion bei den in der Querschnittsleitlinie der Bundesärztekammer (BÄK) zur Therapie mit Blutkomponenten und Plasmaderivaten empfohlenen Hämoglobin(Hb)-Werten sahen (bei stabilem Kreislauf bei <4,3 mmol/l und bei instabilem Kreislauf bei <5,0 mmol/l) [7]. Die Kolinearität der verwendeten Prädiktoren wurde anhand des Variance Inflation Factor (VIF) bewertet, wobei alle VIF-Werte unter 1.3 lagen. Die Punktschätzer (Odds Ratio, OR) einschließlich der zugehörigen 95 %-Konfidenzintervalle (95 %-KI) wurden auf Basis der folgenden Referenzkategorien der Prädiktoren berechnet: „Grund- und Regelversorger“ (Versorgungsstufe), „*n* = 0 bis 3“ (Erfahrung in postmortalen Organspenden), „nicht absolviert“ (TxB-Curriculum) und „nicht vorhanden“ (ZB Intensivmedizin). Die statistische Signifikanz wurde anhand eines mithilfe der Bonferroni-Korrektur adaptierten Signifikanzniveaus von α = 0,005 bewertet. Alle Analysen wurden mit RStudio (Version 2023.12.1.402) durchgeführt [31]. Die Erstellung der Abbildungen erfolgte mittels GraphPad Prism 10 für macOS (Version 10.4.1, GraphPad Software LLC, Boston, MA, USA).

## Ergebnisse

### Demografie

Insgesamt wurden 951 Fragebogen ausgewertet, was einer Rücklaufquote von 8,4 % entspricht. Eine Übersicht über die demografischen Daten der Befragten ist in Tab. 1 dargestellt. Die große Mehrheit (98 %) der Antwortenden waren Fachärztinnen und Fachärzte, die sich auf die unterschiedlichen Versorgungsstufen verteilten (28 % Grund‑/Regelversorger, 29 % Schwerpunktversorger, 17 % Maximalversorger, 24 % Universitätskliniken, 1 % Sonstige). Ein Drittel gab an, TxB seiner Klinik zu sein. Das Vorhandensein eines Standards im Sinne einer Standard Operating Procedure (SOP) zum Thema wurde von 45 % bejaht, währenddessen sich 94 % aller Befragten die Erstellung von Handlungsempfehlungen wünschen.

### Medikamentengabe

Die relativen Häufigkeiten der Gabe der einzelnen anästhesiologisch relevanten Medikamente zeigt Abb. 1. Ein Opioid wird von 72 % der Antwortenden entweder bei jeder oder in über 50 % der Organspenden verwendet, ein Muskelrelaxans von 81 %. Ein volatiles Anästhetikum (in 94 % Sevofluran) wird von 31 % der Befragten in der überwiegenden Anzahl der Organspenden verabreicht, und 22 % wiederum setzen ein i.v.-Hypnotikum ein. Dopamin wird von 63 % nicht verabreicht. Zudem gaben 55 % der Befragten an, bei über 50 % der Organspenden (oder bei jeder) eine PAP zu verabreichen, bezüglich der Gabe eines Glukokortikoids trifft dies auf 46 % zu (alle Abb. 1).

In der logistischen Regressionsanalyse (Abb. 2 und Zusatzmaterial online: ESM 2) zeigte sich, dass Ärztinnen und Ärzte an Kliniken der Maximalversorgung eine signifikant höhere Wahrscheinlichkeit für die Verabreichung von Dopamin, Glukokortikoiden und einer PAP im Vergleich zu Ärztinnen und Ärzten an Kliniken der Grund- und Regelversorgung aufwiesen (Dopamin: OR 2,38 [95 %-KI 1,49–3,78], Glukokortikoide: 2,28 [95 %-KI 1,42–3,65], PAP: 1,93 [95 %-KI 1,23–3,03], Abb. 2). Auch bei Mitarbeitenden an Universitätskliniken ist die OR für die Verabreichung von Glukokortikoiden signifikant höher (1,88 [95 %-KI 1,23–2,88], Abb. 2). Ferner zeigte sich, dass die OR für die Gabe von Dopamin und Glukokortikoiden bei Ärztinnen und Ärzten, die das Curriculum zum TxB absolviert hatten, im Vergleich zu Ärztinnen und Ärzten ohne diese Weiterbildung ebenfalls signifikant erhöht war (Dopamin: 4,67 [95 %-KI 3,38–6,44], Glukokortikoide: 3,37 [95 %-KI 2,38–4,77]), der Einsatz eines i.v.-Hypnotikums wiederum war als TxB weniger wahrscheinlich (0,52 [0,36–0,74]). Die Erfahrung bezüglich einer aktiven Beteiligung an einer Organspende sowie der Erwerb der Zusatzbezeichnung Intensivmedizin hatten keinen Einfluss auf die Wahrscheinlichkeit der Gabe eines der genannten Medikamente (alle Abb. 2).

### Hämodynamisches Management

Mehr als die Hälfte (51 %) der Befragten gaben an, einen mittleren arteriellen Blutdruck von mindestens 65–69 mm Hg während der postmortalen Organspende anzustreben. Als primärer Vasopressor wird Noradrenalin in 97 % der Fälle verwendet. Bezüglich der Transfusionstrigger sahen bei einem kreislaufstabilen Organspender 47 % der Befragten eine Bluttransfusion bei einem Hb-Wert < 4,3 mmol/l (7,0 g/dl) als indiziert an, während 50 % bei Kreislaufinstabilität die Transfusion schon bei einem Wert < 5,0 mmol/l (8,0 g/dl) vornehmen würden. 11 % der Befragten sahen generell keine Indikation zur Transfusion bei einem kreislaufstabilen Organspender. In der Regressionsanalyse konnte kein Zusammenhang zwischen der Versorgungsstufe, der Erfahrung in der Organspende, der Absolvierung des Curriculums oder dem Erwerb der Zusatzbezeichnung Intensivmedizin auf die Indikationsstellung der Transfusion im Sinne der Querschnittsleitlinie der BÄK [7] festgestellt werden (Tab. 3). Ein erweitertes hämodynamisches Monitoring wird von 83 % der Befragten in der überwiegenden Zahl der Organspenden verwendet (Tab. 2).

## Diskussion

Zur Praxis des anästhesiologischen Managements einer postmortalen Organspende in Deutschland existieren bisher keine Daten. Die Ergebnisse der vorliegenden prospektiven Querschnittstudie zeigen deutlich, dass das anästhesiologische Management in den einzelnen Entnahmekrankenhäusern bzw. durch das ärztliche Personal sehr heterogen und individuell gehandhabt wird. Dies könnte mitunter dadurch bedingt sein, dass für diesen Bereich des Transplantationsprozesses bisher keine Handlungsempfehlungen oder Leitlinien existieren [14], wenngleich sich fast alle Befragten in der Umfrage die Erstellung einer solchen Entscheidungshilfe wünschen würden.

Das übergeordnete Ziel des anästhesiologischen Managements zur Organspende ist es, die bestmögliche Qualität der gespendeten Organe sicherzustellen [28]. Hierbei werden jedoch nicht nur die bereits auf der Intensivstation begonnenen Maßnahmen zur Organprotektion fortgesetzt [17, 18], sondern es bieten sich auch Chancen, die Organqualität und damit auch das Behandlungsergebnis der folgenden Transplantation(en) durch spezifische Maßnahmen und Interventionen ggf. günstig zu beeinflussen [14]. Als Beispiel ist hier der Einsatz der volatilen Anästhetika zu nennen: Diese werden im Rahmen der postmortalen Organspende nicht zur Etablierung oder zur Aufrechterhaltung einer Narkose – dies ist nach irreversiblen Hirnfunktionsausfall (IHA), wie vom Deutschen Ethikrat festgestellt, nicht mehr notwendig [12] –, sondern ausschließlich aufgrund ihrer potenziell organprotektiven Eigenschaften verwendet [14, 20]. Obgleich bisher wenig Evidenz zum Einsatz dieser Substanzen während der postmortalen Organspende vorliegt [4, 21], konnte ein positiver Effekt in einigen wenigen klinischen Studien aufgezeigt werden [3, 19]. Diese Ergebnisse führten beispielsweise in der Schweiz zu einer Empfehlung für den Einsatz volatiler Anästhetika während der Organspende [5]. In Deutschland scheint sich dieses Vorgehen jedoch noch nicht durchgesetzt zu haben, denn in der aktuellen Befragung gaben nur 31,6 % an, volatile Anästhetika (vorwiegend Sevofluran) bei jeder (17,3 %) oder zumindest in der überwiegenden Zahl der Organspenden (14,3 %) einzusetzen.

Die Bedeutung des Blutdrucks als entscheidende Determinante für einen suffizienten Perfusionsdruck der einzelnen Organsysteme, insbesondere der Niere, konnte in den letzten Jahren anhand mehrerer großer Patientenkollektive, die sich einem elektiven operativen Eingriff unterziehen mussten, nachgewiesen werden [24, 26]. Wenngleich, wie bereits erwähnt, keine expliziten Handlungsempfehlungen zum hämodynamischen Management während einer Organspende existieren, so streben gemäß der Befragung 79 % einen mittleren arteriellen Blutdruck über 65 mm Hg und 94 % über 60 mm Hg an. Als primäres Medikament zur Anhebung des Blutdrucks wird von 97 % der Befragten Noradrenalin verwendet. Dies deckt sich mit internationalen Empfehlungen sowohl für elektive Operationen als auch für die Organspende [5, 24] und zeigt – insbesondere bezüglich des zu verwendenden Medikaments – den hohen Grad der Verbreitung dieser Vorgaben und der diesen zugrunde liegenden wissenschaftlichen Untersuchungen. Die ebenfalls hohe Rate bezüglich des Einsatzes eines erweiterten hämodynamischen Monitorings ist nicht überraschend, da dies insbesondere für die intensivmedizinische Therapie nach IHA empfohlen wird und somit bei Ankunft im OP bereits etabliert ist [18].

Die Indikationsstellung zur Transfusion von Erythrozytenkonzentraten ist in Deutschland durch die entsprechende Querschnittsleitlinie der BÄK geregelt [7]. Die Organspende ist hier zwar nicht explizit erwähnt, es wird aber empfohlen, sich auch im Rahmen einer organprotektiven Therapie an dieser Leitlinie zu orientieren [17, 18]. Dennoch war die Antwortverteilung in der vorliegenden Untersuchung sehr heterogen, und 11 % der Befragten betrachteten eine Transfusion bei kreislaufstabilen oder -instabilen Spendern unabhängig vom Hb-Wert als nicht indiziert. Dennoch gab jeweils (fast) die Hälfte der Befragten an, dass sie bei den in der Querschnittsleitlinie für stabile und instabile Kreislaufverhältnisse empfohlenen Untergrenzen des Hb-Werts (4,3 bzw. 5,0 mmol/l) auch während einer Organspende transfundieren. Ein Einfluss der untersuchten Faktoren (Versorgungsstufe, Erfahrung und Ausbildung) auf die Adhärenz bezüglich der Transfusionstrigger aus der Leitlinie konnte in der Befragung nicht nachgewiesen werden. Dennoch sollte es das Ziel sein, die Empfehlungen bezüglich der Indikationsstellung der Transfusion während einer Organspende ähnlich effektiv zu verbreiten, wie dies bereits im Bereich des hämodynamischen Managements der Fall zu sein scheint.

Und auch wenn der Einfluss einer Weiterbildung im Hinblick auf die Transfusion nicht gezeigt werden konnte, kommt der Edukation des ärztlichen und pflegerischen Personals eine entscheidende Bedeutung zu, um die generellen Konzepte, Chancen und Risiken rund um das anästhesiologische Handeln während einer Organspende flächendeckend zu verbreiten und eine entsprechend hohe Durchdringung zu erreichen [30]. Aus den Ergebnissen der vorliegenden Studie wird deutlich, dass Ärztinnen und Ärzte, die bereits das Curriculum zum TxB durchlaufen haben, mitunter andere Entscheidungen während der postmortalen Organspende treffen als ohne absolviertes Curriculum. Beispielsweise applizieren darin geschulte Ärztinnen und Ärzte signifikant seltener ein i.v.-Hypnotikum während einer Organspende, setzen dafür aber umso häufiger ein Glukokortikoid ein. Das Curriculum zum TxB basiert auf einem von der BÄK vorgegebenen Lehrplan, in dem explizit auf alle wesentlichen Aspekte der Organspende eingegangen wird. Dazu gehören u. a. das Konzept des IHA sowie die Auswirkungen des medizinischen Handelns, einschließlich der Gabe verschiedener Medikamente, auf die Organprotektion [6].

Obwohl die vorliegenden Ergebnisse einen Effekt des Curriculums auf das anästhesiologische Handeln aufzeigen, erreicht diese Weiterbildung nicht alle entsprechenden Ärztinnen und Ärzte in den deutschlandweit aktuell 1132 Entnahmekrankenhäusern [10]. Eine entsprechende Handlungsempfehlung oder eine Leitlinie hätte hier wahrscheinlich ein deutlich größeres Potenzial, die entsprechenden Inhalte einer breiten Öffentlichkeit zu vermitteln und so nicht nur als Entscheidungshilfe zu wirken, sondern auch die Behandlungs- und damit auch die Organqualität insgesamt zu verbessern [18].

Auch die Versorgungsstufe des Krankenhauses, in dem die Organentnahme erfolgt, scheint sich auf das anästhesiologische Handeln auszuwirken: In der Regressionsanalyse konnte klar gezeigt werden, dass anästhesiologisches ärztliches Personal, welches an Maximalversorgern und Universitätskliniken arbeitet, mit einer höheren Wahrscheinlichkeit Glukokortikoide oder Dopamin verabreicht als das ärztliche Personal an Kliniken der Grund- und Regelversorgung. International wird die Gabe von Glukokortikoiden v. a. bei kreislaufinstabilen Spenderinnen und Spendern im Rahmen der intensivmedizinischen Therapie empfohlen, wenngleich die korrekte Dosierung unklar ist [14]. Die DSO empfiehlt in ihrem Leitfaden ebenfalls die Gabe [17, 18].

Die Applikation von Dopamin in niedriger Dosierung (4 µg·kg⁻¹·min⁻¹) wird seit 2017 von der Deutschen Transplantationsgesellschaft [11] und wurde zwischenzeitlich (2019 bis 2025) auch von der DSO empfohlen [9]. Die Evidenz ist jedoch begrenzt, da nur sehr wenige Studien einen möglichen positiven Effekt der Dopamingabe vor der Organspende auf die Frühfunktion des Organs beispielsweise nach einer Nierentransplantation bei Erwachsenen [25] oder nach Herztransplantation im Kindesalter [22] zeigen konnten. In beiden Studien hatte jedoch die Dopamingabe keinen Einfluss auf die Langzeitfunktion oder das Gesamtüberleben der transplantierten Personen [22, 25]. Eine kürzlich veröffentlichte Metaanalyse von 7 Studien (*n* = 767) kam zudem zum Schluss, dass nach aktueller Datenlage nicht von einem positiven Effekt der Gabe von Dopamin nach einer Nierentransplantation auszugehen ist [33]. In den 3 analysierten Studien (*n* = 547), bei denen das Medikament bereits vor der Organentnahme verabreicht wurde, konnte kein Unterschied hinsichtlich des Risikos für eine akute Tubulusnekrose, des Transplantatüberlebens oder der Sterblichkeit bei den organempfangenden Personen nachgewiesen werden. Vier weitere Studien, die einen möglichen Effekt der Gabe von Dopamin nach der Transplantation untersuchten, konnten in der Metaanalyse ebenfalls keinerlei Auswirkungen auf das Behandlungsergebnis zeigen, wenngleich die Vergleichbarkeit der Studien aufgrund unterschiedlicher Endpunkte sicherlich eingeschränkt ist [33]. Zudem wurden im Bereich der Lungentransplantation auch negative Auswirkungen einer Dopamingabe im Sinne eines schlechteren Transplantatüberlebens berichtet [29]. Aufgrund der aktuellen Evidenzlage wird daher in der Leitlinie zum Spendermanagement in Kanada von einer Dopamingabe konkret abgeraten [2]. In der aktuellen Umfrage gaben 63 % der Befragten an, das Medikament nicht einzusetzen. Die Gründe hierfür wurden jedoch nicht erfragt und bleiben daher unklar.

Die Gabe einer PAP im Rahmen der Organentnahme wird gemäß den Umfrage-Ergebnissen von Ärztinnen und Ärzten sehr uneinheitlich gehandhabt, was sich mit Ergebnissen einer ähnlichen Untersuchung in Frankreich deckt [8]. Die eigentliche Indikation für eine PAP, d. h. die Vermeidung von postoperativen Wundinfektionen, scheint auf den ersten Blick für die Organspende irrelevant zu sein. Andererseits muss eine Infektion der organempfangenden Person unbedingt vermieden werden, was sich in der Empfehlung für eine PAP während der Transplantation widerspiegelt [1]. Generell gibt es bisher wenig Evidenz und Empfehlungen zum antiinfektiven Management von organspendenen Personen und der Relevanz einer PAP während der Entnahme [27]. Eine kürzlich veröffentlichte Studie zum Thema konnte zudem zeigen, dass die Gabe einer PAP während der Organentnahme mit 21,5 % deutlich seltener vorgenommen wurde als eine antimikrobielle Therapie zuvor auf der Intensivstation (76,5 %) [13]. Dies zeigt einmal mehr, dass es weiterer Studien zum (anästhesiologischen und intensivmedizinischen) Management der Organspende bedarf, um auch die Relevanz einer PAP zu klären und evidenzbasierte Empfehlungen abgeben zu können.

### Limitationen

Die Rücklaufquote der Umfrage betrug 8,4 %. Die Interpretation der Ergebnisse sollte daher nur unter Berücksichtigung einer möglichen Selektionsverzerrung vorgenommen werden, und Rückschlüsse auf die Gesamtheit der Anästhesiologinnen und Anästhesiologen in Deutschland sind sicherlich nicht möglich. Es darf aber durchaus spekuliert werden, dass v. a. diejenigen geantwortet haben, die die vorliegende Thematik unmittelbar betrifft: So gaben 95 % der Befragten an, mindestens eine und 67 % bereits mehr als 4 Organspenden selbst begleitet zu haben, sodass die Annahme eines repräsentativen Ergebnisses für die aktiv in Organspenden eingebundenen Personen durchaus gerechtfertigt sein könnte.

Es bleibt zudem unklar, inwieweit sich die Tatsache, dass in 23 % der betreffenden Kliniken im Jahr vor der Erhebung keine Organspende durchgeführt wurde, auf die Antworten und damit auch auf die Qualität der Versorgung ausgewirkt haben könnte. Aus anderen Untersuchungen ist bekannt, dass sich die Erfahrung der handelnden Personen insbesondere im Bereich des anästhesiologischen Managements maßgeblich auf das Behandlungsergebnis nach einer Operation auswirken kann [23]. Bei einem seltenen Ereignis wie einer Organspende könnte sich dieser negative Effekt bei einem unerfahrenen Team dann ggf. sogar noch potenzieren [23].

## Fazit

- Zusammenfassend kann festgestellt werden, dass das anästhesiologische Management der postmortalen Organspende in Deutschland aktuell sehr unterschiedlich gehandhabt wird.
- Die vorliegende Studie stellt dabei die erste wissenschaftliche Untersuchung zum Thema in Deutschland dar. In der Analyse konnte gezeigt werden, dass sowohl die Versorgungsstufe des Entnahmekrankenhauses als auch die Edukation des ärztlichen Personals sich auf die entsprechenden anästhesiologischen Interventionen auswirken könnten.
- Die Erstellung von Handlungsempfehlungen wiederum könnte einen ersten Schritt zur Vereinheitlichung des anästhesiologischen Handelns darstellen.
- Eine solche Empfehlung würde aufgrund der begrenzten wissenschaftlichen Evidenz sicherlich hinsichtlich ihrer Aussagekraft bezüglich des Behandlungsergebnisses limitiert sein.
- Für die Zukunft besteht daher ein erheblicher Forschungsbedarf zur Optimierung des anästhesiologischen Handelns im Hinblick auf eine bestmögliche Organprotektion während der Organspende.
- Dennoch könnte durch eine Vereinheitlichung die Sicherheit der handelnden Personen bezüglich des anästhesiologischen Managements einer Organspende und damit auch Qualität der Versorgung weiter verbessert werden.

## Supplementary Information

ESM 1_Rest der Umfrage

ESM 2_Regression

## Data Availability

Die in dieser Studie erhobenen Datensätze können auf begründete Anfrage beim Korrespondenzautor angefordert werden.
