# Supplementary material for: Perioperative anesthesiological management of postmortem organ donors in Germany—A prospective cross-sectional study using an online survey
Source: Anaesthesiologie. 2026 Jan 28;75(3):175–84. [Article in German] doi: 10.1007/s00101-026-01647-5 (PMC12917051; doi:10.1007/s00101-026-01647-5)
Supplement: Supplementary file 1 — ESM 1_Rest der Umfrage [file 101_2026_1647_MOESM1_ESM.pdf]

Zusatzmaterial zum Beitrag „Perioperatives anästhesiologisches Management der postmortalen Organspende in Deutschland – Eine Online-Umfrage unter den Mitgliedern der Deutschen Gesellschaft für Anästhesiologie und Intensivmedizin“ von T. Piegeler, J.S. Englbrecht, M. Söhle et al. (2026) in *Die Anaesthesiologie.*

Beitrag und Zusatzmaterial stehen Ihnen auf www.springermedizin.de zur Verfügung. Bitte geben Sie dort den Beitragstitel in die Suche ein.

Weitere im Rahmen der Studie abgefragte Kategorien bzgl. des anästhesiologischen Managements der postmortalen Organspende

Frage	Anteil [%]	N
ORGANISATORISCHES		
An der Klinik der Befragten durchgeführte postmortale **Organentnahmen im Jahr 2022**		932
· 0	23	
· 1 - 4	42	
· 5 - 10	16	
· 11 - 20	6	
· > 20	3	
· Keine Angabe/weiss nicht	10	
Vorhandensein eines **Standards**/einer **SOP** zum Thema (ja / nein)	45 / 55	949
Wunsch nach einer **Handlungsempfehlung** zum Thema "Anästhesiologische Begleitung der postmortalen Organspende" (ja / nein)	94 / 6	947
ZUSATZFRAGEN MEDIKAMENTENGABE		
**Am häufigsten verabreichtes OPIOID**		733
· Sufentanil	77	
· Fentanyl	20	
· Remifentanil	3	
· Alfentanil	0,1	
· Anderes	0,1	
**Am häufigsten verabreichtes MUSKELRELAXANS**		833
· Rocuronium	72	
· Vecuronium	0,2	
· (Cis-)Atracurium	24	
· Pancuronium	4	
· Anderes	0,3	
**Am häufigsten verabreichtes HYPNOTIKUM**		259
· Propofol	87	
· Thiopental	2	
· Etomidate	0,4	
· Midazolam	11	
· Anderes	0	

**Am häufigsten verwendeter Applikationsweg für HYPNOTIKUM**		255
· Bolus	20	
· Kontinuierlich via Perfusor	76	
· Kontinuierlich via target-controlled infusion (TCI)	4	
**Am häufigsten verabreichtes VOLATILES ANÄSTHETIKUM**		406
· Sevofluran	94	
· Desfluran	3	
· Isofluran	3	
· Anderes	0	
**Am häufigsten verabreichtes GLUCOCORTICOID**		613
· Hydrocortison	68	
· Dexamethason	8	
· (Methyl-)Prednisolon	24	
· Anderes	0	
**Am häufigsten verabreichte PAP**		591
· Cephalosporine	61	
· (Amino-)Penicilline	36	
· Carbapeneme	2	
· Glykopeptide	0,2	
· Aminoglykoside	0	
· Fluorchinolone	0,2	
· Anderes	1,4	

SOP = (engl.) Standard Operating Procedure, PAP = perioperative Antibiotika-Prophylaxe
