# Supplementary material for: Perioperative anesthesiological management of postmortem organ donors in Germany—A prospective cross-sectional study using an online survey
Source: Anaesthesiologie. 2026 Jan 28;75(3):175–84. [Article in German] doi: 10.1007/s00101-026-01647-5 (PMC12917051; doi:10.1007/s00101-026-01647-5)
Supplement: Supplementary file 2 — ESM 2_Regression [file 101_2026_1647_MOESM2_ESM.pdf]

Zusatzmaterial zum Beitrag „Perioperatives anästhesiologisches Management der postmortalen Organspende in Deutschland – Eine Online-Umfrage unter den Mitgliedern der Deutschen Gesellschaft für Anästhesiologie und Intensivmedizin" von T. Piegeler, J.S. Englbrecht, M. Söhle et al. (2026) in *Die Anaesthesiologie.*

Beitrag und Zusatzmaterial stehen Ihnen auf www.springermedizin.de zur Verfügung. Bitte geben Sie dort den Beitragstitel in die Suche ein.

Ergebnisse der multiplen logistischen Regressionsanalyse

Medikament / Einflussfaktor [OR (95%KI)]	Opioid	Muskelrelaxans	Hypnotikum	Volatiles Anästhetikum	Glucocorticoid	PAP	Dopamin
Versorgungsstufe							
Schwerpunktversorger	0,81 (0,53-1,24)	0,87 (0,50-1,53)	0,71 (0,47-1,05)	1,08 (0.76-1,54)	1,27 (0,87-1,86)	1,28 (0,89-1,84)	1,54 (1,04-2,27)
Maximalversorger	0,91 (0,54-1,52)	1,12 (0,56-2,24)	0,57 (0,34-0,93)	0,80 (0,52-1,23)	***2,28 (1,42-3,65)**	***1,93 (1,23-3,03)**	***2,38 (1,49-3,78)**
Universitätsklinikum	0,88 (0,55-1,42)	1,09 (0,58-2,05)	0,87 (0,57-1,34)	1,27 (0,86-1,88)	***1,88 (1,23-2,88)**	1,50 (1,00-2,25)	1,58 (1,02-2,46)
Erfahrung							
n = 4 bis 10	1,25 (0,83-1,87)	1,17 (0,71-1,92)	1,01 (0,71-1,44)	0,76 (0,55-1,05)	0,96 (0,67-1,38)	0,86 (0,61-1,21)	1,07 (0,74-1,53)
n > 10	0,86 (0,56-1,33)	1,47 (0,80-2,70)	0,60 (0,39-0,93)	0,80 (0,56-1,16)	0,61 (0,41-0,91)	0,80 (0,54-1,18)	1,06 (0,71-1,59)
Curriculum TxB	0,83 (0,58-1,18)	1,78 (1,06-2,98)	***0,52 (0,36-0,74)**	0,98 (0,73-1,32)	***3,37 (2,38-4,77)**	1,26 (0,93-1,72)	***4,67 (3,38-6,44)**
Zusatzbezeichnung Intensivmedizin	1,19 (0,79-1,80)	1,30 (0,78-2,15)	0,64 (0,45-0,92)	1,10 (0,78-1,55)	1,64 (1,15-2,35)	1,51 (1,06-2,14)	1,06 (0,72-1,55)

OR = Odds ratio, KI = Konfidenzintervall, TxB = Transplantationsbeauftragter.
Verwendete Referenzkategorien: Versorgungsstufe: Grund- und Regelversorger, Erfahrung Organspende: n = 0 bis 3, Curriculum: Nicht vorhanden/absolviert, Zusatzbezeichnung Intensivmedizin: Nicht vorhanden/absolviert. * p < 0,005.
